# Pulmonary papillary adenoma with malignant potential: a case report and literature review

**DOI:** 10.1186/s13000-022-01259-8

**Published:** 2022-10-13

**Authors:** Ping Liu, Junjian Feng, Min Yang, Jingqiu Chen, Luyao Fu, Junxu Lu

**Affiliations:** 1 Department of Pathology, Luzhou People’s Hospital, Jiangyang District, 646000 Luzhou, Sichuan Province People’s Republic of China; 2 Department of Intensive Care Unit, Luzhou People’s Hospital, Section 2 of Jiugu Avenue, Jiangyang District, 646000 Luzhou City, Sichuan Province People’s Republic of China; 3 Department of Medical Imaging, Luzhou People’s Hospital, Jiangyang District, 646000 Luzhou, Sichuan Province People’s Republic of China

**Keywords:** Pulmonary papillary adenoma, Peripheral tumor, Alveolar adenoma, Lung tumor, Malignant potential

## Abstract

**Background:**

Pulmonary papillary adenoma is a rare benign tumor in the periphery of the lung. We report a 66-year-old female patient with a tumor in the lower lobe of the right lung and present the clinicopathological features and review the literature.

**Case presentation:**

A tumor in the lower lobe of the right lung was found incidentally on chest X-ray during the physical examination of the patient, and the patient occasionally had a dry cough that was not treated. The tumor was clearly demarcated and lobulated on CT scan. After 2 years of follow-up, the boundary of the tumor was still clear, with more lobulations and the enhanced scan showed uniform enhancement. Grossly, the tumor had a granular cut surface and was easy to fall off, which was helpful for the diagnosis of papillary adenoma during intraoperative frozen examination. Under the microscope, most areas of the tumor had the typical morphological structure of papillary adenoma. However, the tumor locally protruded into the surrounding lung tissue, accompanied by crowded cells and high cell proliferation index. It was suggested that this case of papillary adenoma had malignant potential and needed active intervention and treatment.

**Conclusion:**

Pulmonary papillary adenoma is a rare epithelial tumor with malignant potential. Surgical treatment should be performed as soon as possible after diagnosis to prevent malignant transformation.

**Supplementary Information:**

The online version contains supplementary material available at 10.1186/s13000-022-01259-8.

## Background

Pulmonary papillary adenoma is a rare benign epithelial tumor of the lung, which often occurs in the periphery of the lung. Spencer et al. [1] first reported it in 1980, and 41 cases have been reported worldwide so far. We reported another case of a papillary adenoma with malignant potential and reviewed the literature to deepen our understanding of the tumor.

## Case Presentation

### Clinical history

**Fig. 1:**
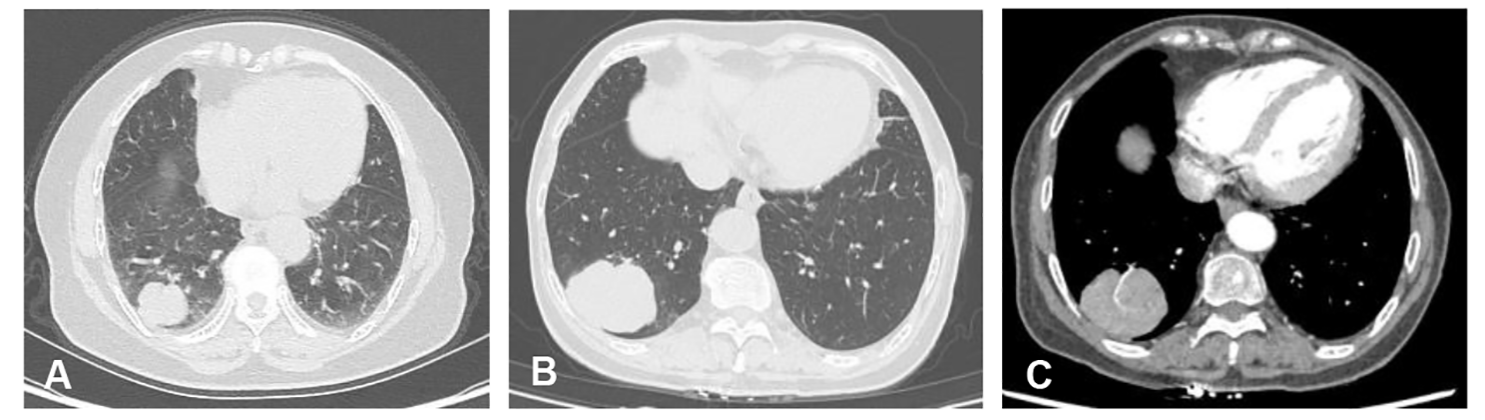
The chest CT scan manifestation of the tumor. A, In 2018, the tumor was well circumscribed, lobulated, and the maximum diameter was 3.7 cm. B, In 2021, the maximum diameter of the mass was 5.5 cm, with more obvious lobulation. C, Blood vessel was visible in the arterial phase of the tumor

A 66-year-old female patient underwent a chest X-ray during the physical examination and incidentally found a mass in the lower lobe of the right lung 2 years ago. The patient was previously healthy and had no history of smoking, chest pain, dyspnea and other symptoms, but occasionally dry cough. The chest CT scan (November 17, 2018) showed an irregular soft tissue mass in the posterior basal segment of the lower lobe of the right lung, with a size of about 3.7 cm × 3.4 cm × 2.6 cm, with visible lobulation and clear boundary (Fig. 1 A). The patient did not receive treatment. On May 10, 2021, re-examination of chest CT revealed that the volume of the right lower lobe mass was larger than that in 2018, and the size was about 5.5 cm×5.2 cm×4.2 cm. The mass was well-bounded and lobulated without pleural traction (Fig. 1B). The enhanced scan showed uniform and continuous enhancement of the lesion, and the CT value of each phase was between 75HU and 85HU, and blood vessel was visible in the arterial phase of the tumor (Fig. 1 C). Bilateral hilar and mediastinal structures were clear without enlarged lymph nodes. Peripheral lung cancer was considered in imaging.

### Gross features and intraoperative frozen examination

**Fig. 2:**
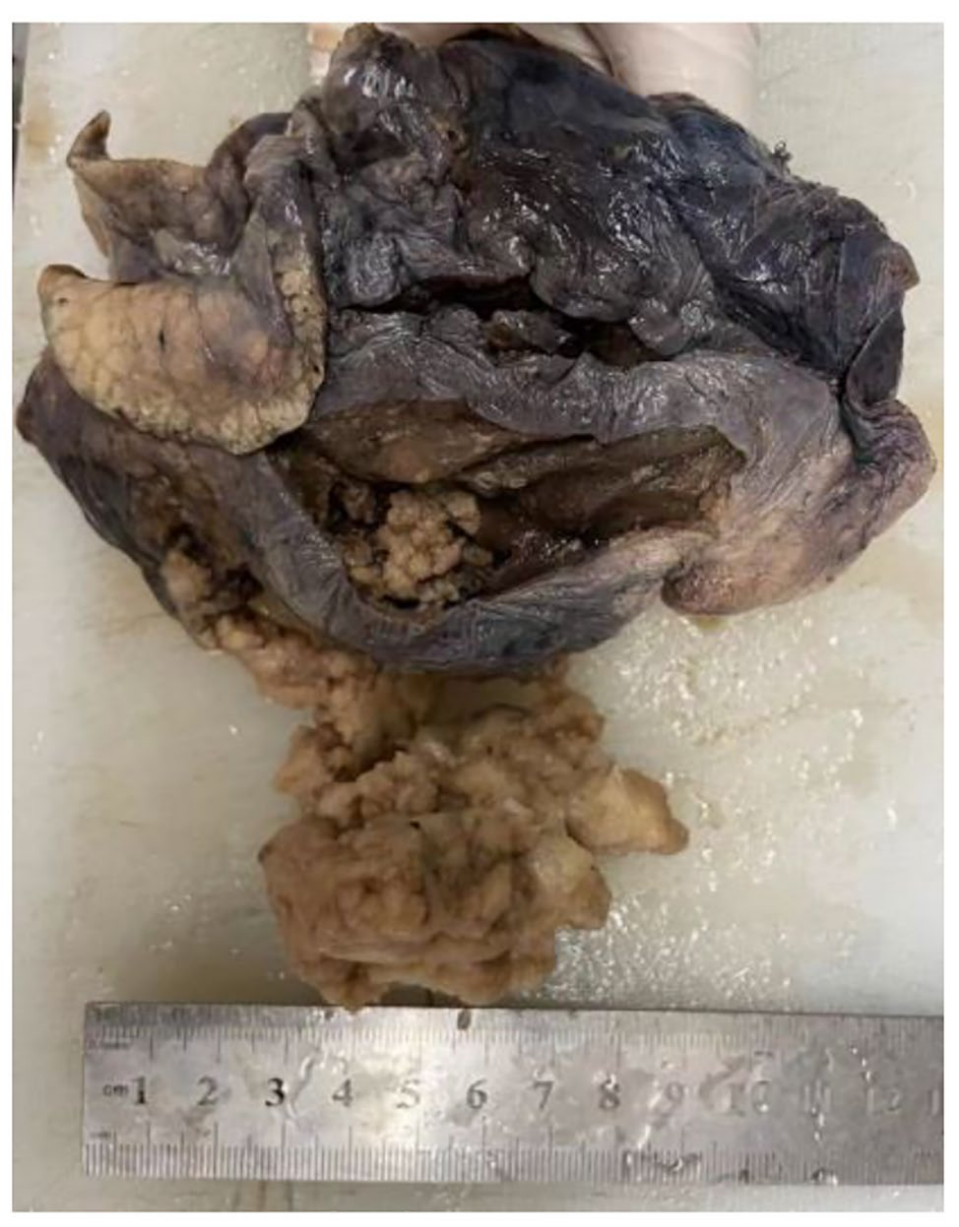
Grossly, the cut surface of the tumor was gray white, solid and granular, without an envelope, and has a clear boundary with the surrounding lung tissue

The tumor was located in the anterior segment of the upper lobe of the right lung. The surgeon removed the tumor under thoracoscopy and performed an intraoperative frozen examination. Gross examination revealed a 5.0 cm×4.5 cm×4.0 cm mass in the stump of the partially resected lung without an envelope and has a clear boundary with the surrounding lung tissue. The cut surface of the tumor was gray white, solid and granular (Fig. 2). Moreover, the granular tissue was easy to fall off when the tumor was incised. Under microscope, the alveolar epithelial cell-like tumor cells formed a wide range of papillary structures with a fibrous vascular core. The surface of the papilla was covered with a single layer of cubic to columnar epithelium, cilia were visible, and the tumor cells were slightly atypical. After discussion by all pathologists, the final intraoperative pathological diagnosis was pulmonary papillary adenoma. Due to the large tumor, wedge resection was easy to damage the lower pulmonary artery, vein and bronchial trunk, so the right lower lobe resection was performed. In order to avoid the limitation of frozen sampling leading to the upgrading of pathological diagnosis after freezing, two groups of regional lymph nodes were cleaned up.

## Materials and methods

The surgical specimens were fixed in 10% buffered formalin, embedded in paraffin and serially sectioned into 4-µm-thick slices. Routine staining with hematoxylin and eosin (HE) was performed. Representative tissue blocks were also selected for immunohistochemical staining, all antibodies were purchased from Fuzhou Maixin Company. Primary antibodies included anti-CK20, CK7, TTF-1, NapsinA, Tg, P63, P40, WT-1, Ki67 and P53 antibodies.

## Results

### Histologic features

**Fig. 3:**
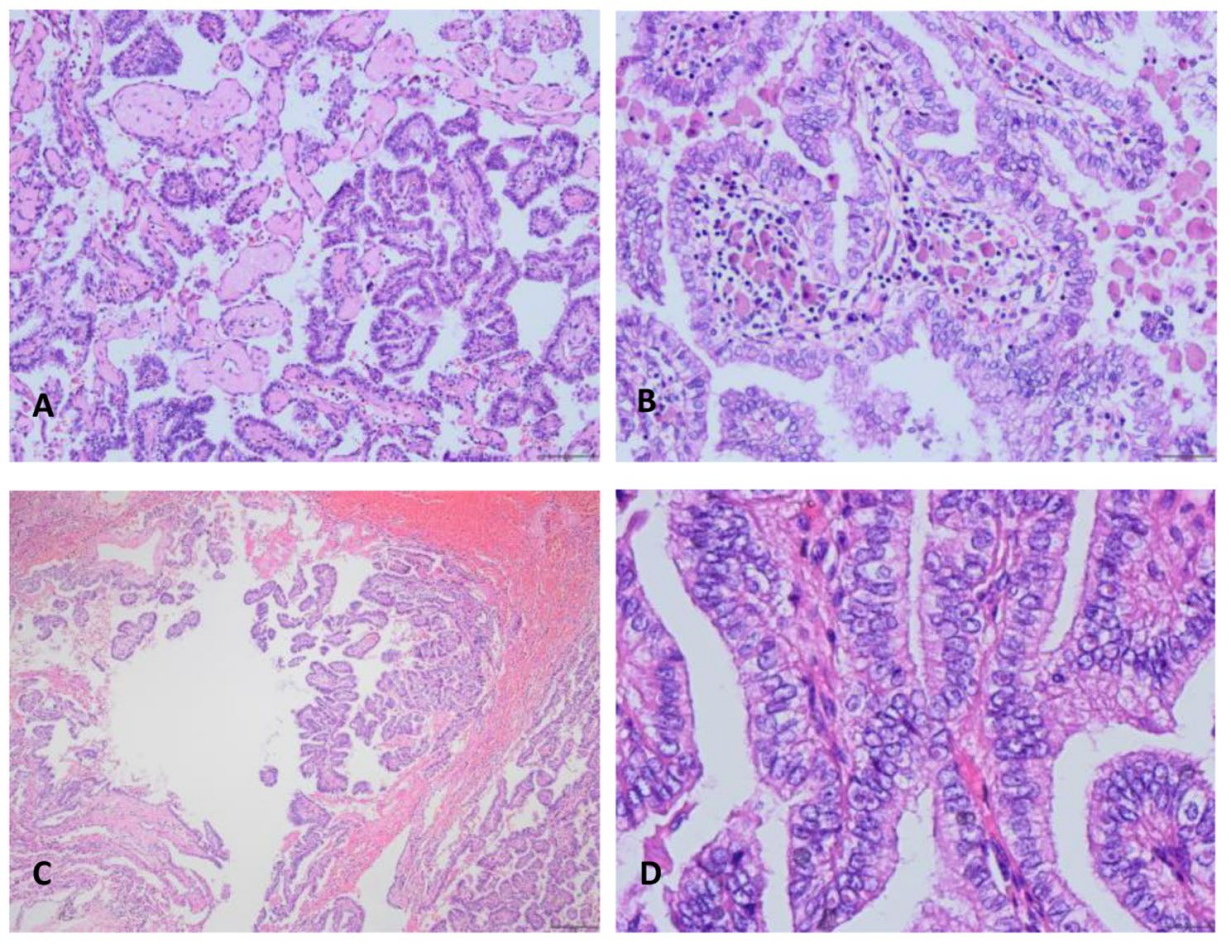
Morphological features of the tumor. A, The tumor was composed of branched papillae with fibrovascular cores, and partial papillary cores hyalinized (100×). B, The fibrovascular core was infiltrated with lymphocytes and histiocytes, cilia can be seen (200×). C, The tumor protruded into the surrounding lung tissue in a mushroom-shaped without pro-fibrointerstitial reaction around (40×). D, The tumor cells were crowded and arranged pseudostratified with obvious nucleoli (400×)

Histologically, the tumor was composed of branched papillae with fibrovascular core, and covering a single layer of cubic to columnar epithelium. The fibrovascular cores were infiltrated with lymphocytes, and few cores hyaline degeneration. And histiocytes were present inside and outside of the papillary structures (Fig. 3 A 3B). Cilia can be seen on the luminal ​surface of these epithelial cells. The cytoplasm of tumor cells with uniform size was eosinophilic or clear, round or oval nucleus. Small nucleoli can be seen in the nucleus, and eosinophilic inclusion bodies can be seen in a few nuclei. No necrosis or mitosis was observed. However, the tumor locally protruded into the surrounding lung tissue in a mushroom-shaped without pro-fibrointerstitial reaction around (Fig. 3 C). In addition, the tumor cells in some areas were crowded and arranged pseudostratified with obvious nucleoli (Fig. 3D). No tumor cell metastasis was observed in regional lymph nodes.

#### Immunohistochemical staining

**Fig. 4:**
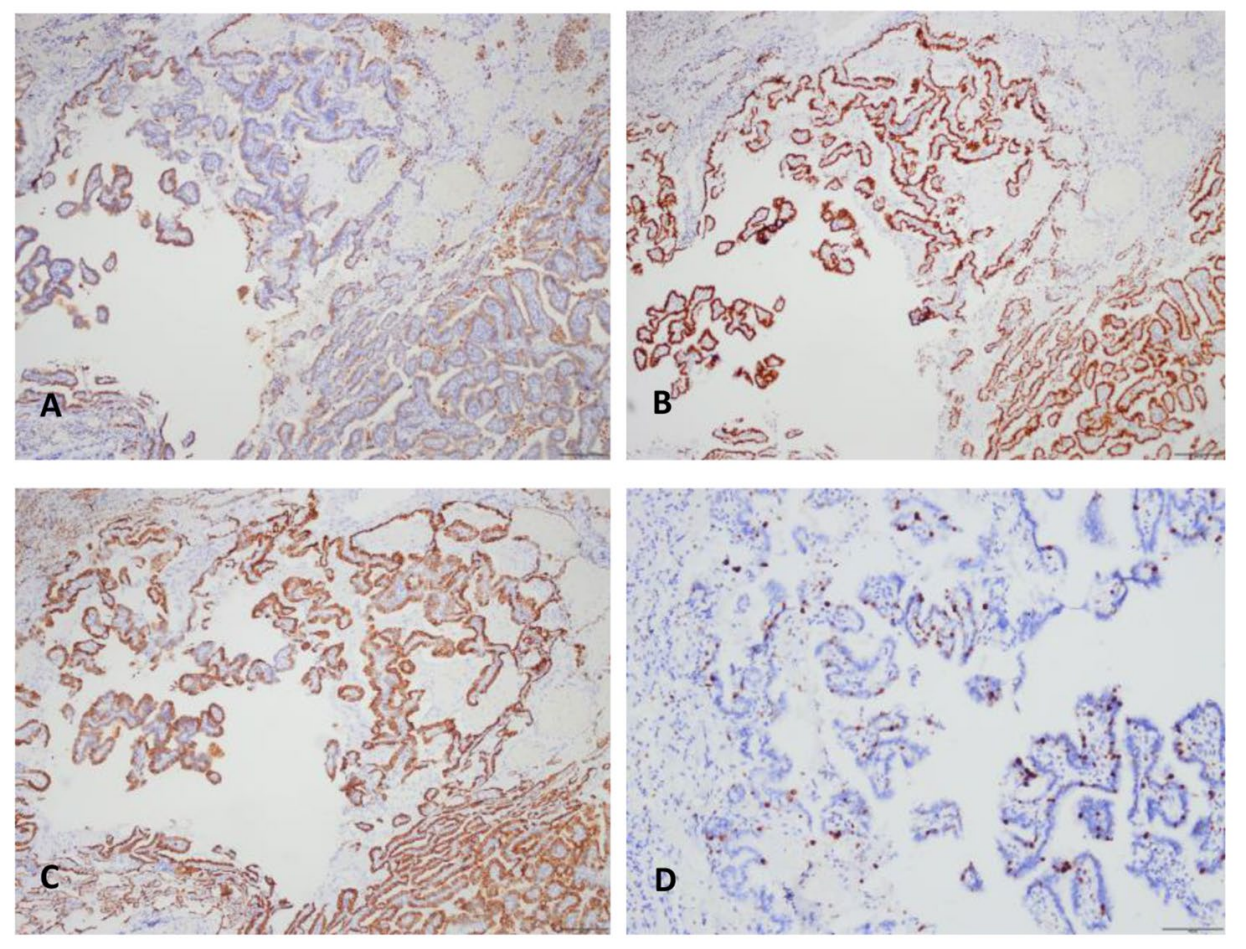
Immunohistochemical staining of the tumor. A, B, C, The tumor cells were diffusely positive for NapsinA, TTF-1, and CK7 (40×). D, Ki-67 proliferative index was about 6%, and even close to 20% in hot spots (100×)

Immunohistochemical staining showed that the tumor cells were diffusely positive for NapsinA, TTF-1, and CK7 (Fig. 4 A 4B 4 C), and negative for Tg, WT-1, P63, P40, and CK20, and few cells showed a weak P53 staining. The Ki-67 proliferative index was about 6%, which was higher than 2% ~ 3% reported in the literature, and even close to 20% in hot spots (Fig. 4D). According to the morphological and immunohistochemical, we considered this to be a case of pulmonary papillary adenoma with malignant potential.

## Discussion

Pulmonary papillary adenoma is a very rare benign epithelial tumor of the lung. So far, 32 cases were reported in English, 9 cases were reported in Chinese, and 1 case was added in this study. There is no conclusive conclusion about its pathogenesis. Many scholars have found that both morphology and osmophilic lamellar bodies or electron-dense particles contained in tumor cells were similar to Clara cells and type II alveolar cells [1–4], so they considered that it originated from pluripotent stem cells of bronchioloalveolar epithelium with the potential to differentiate into Clara cells and type II alveolar cells, and may undergo malignant transformation [5–8].

Patients were usually asymptomatic and most cases were discovered accidentally during physical examinations. Majority of the patients were male and ranged in age from 2 months to 78 years old. Tumors mostly occurred in the periphery of the lung and also in the hilar region [9], usually with single nodule and occasionally with multiple lesions [10]. The most common location was the lower lobe of the left lung, followed by the upper and lower lobes of the right lung. Only a few patients have a history of smoking, indicating that the occurrence of tumors was not related with smoking [11].

Under imaging examination, pulmonary papillary adenomas were mostly solitary round or spherical nodules with smooth margins in the periphery of the lung. In some cases, the edges were slightly rough due to malignant transformation of tumors [7]. The lesions showed lobulation without burr sign, pleural traction or depression, and the enhancement scan showed continuous enhancement [12]. Therefore, it was difficult to differentiate from sclerosing lung cell tumor, alveolar adenoma, and early lung adenocarcinoma on imaging, and the diagnosis depended on pathological examination. In our case, a tumor was discovered in the lower lobe of the right lung in 2018, and the maximum diameter of the tumor increased by about 2 cm after 2 years of follow-up, which still had a clear boundary, but there were more lobulated and passing blood vessels. In enhanced CT scan, the lesions showed uniform continuous enhancement and lobulation, which led the radiologist to consider peripheral lung cancer.

Pathologic examination showed that the diameter of tumors ranged from 0.2 to 9.5 cm, most of which had no capsule and were clearly demarcated from surrounding lung tissues. Under the microscope, the tumor was composed of a large number of branched papillary structures with a fibrous vascular core in the center of the papilla. Lymphocytes, plasma cells and histiocytes cell infiltration and hyaline degeneration can be seen in the cores, as in our case [13]. The papilla was covered with cubic or columnar epithelium, some of which have cilia [14, 15]. The cytoplasm of most tumors was eosinophilic, a few cells were clear and there was no mucus in the cells. The nucleus was usually arranged perpendicular to the basal surface of the cell with small nucleoli, and eosinophilic inclusions in a few nuclei [12]. The tumor cells were mild without mitosis and necrosis. When the tumor underwent malignant transformation, the cell atypia was significantly increased, and the polarity disorder, hyperchromatic nucleus, necrosis and nuclear division were easy to see [8, 16].

Because pulmonary papillary adenomas were extremely rare, pathologists did not have sufficient knowledge of the histological morphology of the tumor. In particular, it was easy to be confused with well-differentiated papillary lung adenocarcinoma, sclerosing lung cell tumor dominated by papillary structure and glandular papilloma during intraoperative frozen examination. In the literature, 2 patients underwent intraoperative frozen examination [10, 17], and 1 case was accurately diagnosed as pulmonary papillary adenoma based on its special gross appearance [17]. The diagnosis rate was low, so it is particularly important to strengthen pathologists’ understanding of this tumor from the aspect of morphology.

Immunohistochemical staining showed that tumor cells were diffusely positive for TTF-1, CK7, NapsinA and other alveolar epithelial markers, and negative for CK5/6, P63 and neuroendocrine markers. Tumor cell proliferation index was usually low, about 2–3% [16], and could be as high as 25–30% when malignant transformation occurred [7, 16]. However, no gene mutation related to adenocarcinoma such as EGFR, K-ras or P53 gene was found by molecular detection regardless of whether the tumor had malignant transformation [7, 9, 18].

Most tumors showed a benign process of slow growth. The treatments were mostly segmental resection or wedge resection, and a few were lobectomy [16, 19]. The patients had a good prognosis and were followed up 6 months to 10 years without recurrence and metastasis [2, 3, 10]. A few literatures reported that tumors invaded the surrounding lung tissue, visceral pleura and venules [1, 5–8, 11]. One of the patients developed acinar adenocarcinoma and micropapillary adenocarcinoma components in the same tumor after 2 years of follow-up [7], which suggested that pulmonary papillary adenoma had malignant potential and should be detected and treated early after diagnosis.

## Conclusion

In summary, pulmonary papillary adenoma is an extremely rare benign epithelial tumor occurring in the periphery of the lung. The typical structure is a single layer of cubic or columnar epithelium on the surface of the papilla with a fibrous vascular core. It is easily to be confused with sclerosing lung cell tumor and well-differentiated papillary adenocarcinoma by imaging and morphology, especially during intraoperative frozen section examination and percutaneous biopsy. Therefore, it is necessary to combine the patient’s clinical data, imaging examination, pathological tissue morphology and immunophenotype comprehensive judgment before diagnosis to avoid misdiagnosis and mistreatment. At present, only a few reports support that the tumor has malignant potential. However, the molecular mechanism of this tumor is unclear, and the common gene mutations of lung adenocarcinoma have not been detected in this tumor. It needs more studies to explain these questions in the future.

## Electronic supplementary material

Below is the link to the electronic supplementary material.

Supplementary Material 1

Supplementary Material 2

## Data Availability

All data generated or analysed during this study was included in this published article.

## Abbreviations

CT: Computed tomography
HE: Hematoxylin and eosin
CK7: Cytokeratin7
CK20: Cytokeratin20
EGFR: Epidermal growth factor receptor
Tg: Thyroglobulin
TTF-1: Thyroid transcription factor 1
WT1: Wilm’s tumor gene
