# Supplementary material for: Pulmonary papillary adenoma with malignant potential: a case report and literature review
Source: Diagn Pathol. 2022 Oct 13;17:81. doi: 10.1186/s13000-022-01259-8 (PMC9563795; doi:10.1186/s13000-022-01259-8)
Supplement: Supplementary file 1 — Supplementary Material 1 [file 13000_2022_1259_MOESM1_ESM.pdf]

泸州市人民医院科研伦理审查申请表

Application Form for Scientific Research Ethics Review of Luzhou People's Hospital

批件号 Reference Number：2012008018

项目名称<br>Project name	Pulmonary papillary adenoma with malignant potential: a case report and literature review
立项依据<br>Basis of project	Rare case report
研究目的<br>Research objective	To investigate the clinicopathological features of pulmonary papillary adenoma.
技术路线<br>Technical route	The clinical and imaging manifestations, histopathological features and immunophenotype of a case of pulmonary papillary adenoma were observed, and the relevant domestic and foreign literatures were reviewed.
受试者类型<br>Subject type	☑住院病人 Inpatient ☐门诊病人 Outpatient<br>☐健康志愿者 Healthy people
受试者例数<br>Number of subjects	1
入选标准及排除标准<br>Inclusion criterion and exclusion criterion	None
所需研究和收集的具体材料[信息、体液(ml、次数)、血液(ml、次数)、组织等]<br>Specific materials to be studied and collected [information, body fluids (ml, times), blood (ml, times), tissue, etc.]	Clinical information and surgical resection specimens of the patient.
检测实验室名称及地点<br>Name and location of laboratory	Department of Pathology, Luzhou People's Hospital

获取材料的方法（手术切除标本、静脉穿刺、腹腔穿刺、住院病史摘录等）<br>Methods of obtaining materials (Surgery, venipuncture, abdominal puncture, medical records, etc.)	surgical resection specimen and hospitalization record		
研究数据处理（统计方法）<br>Statistical analysis	None		
研究项目的预期进度和完成日期<br>Research progress and completion date	October 23, 2021		
申请科室<br>Department of Applicant	Department of Pathology	申请人（签名）<br>Applicant	刘萍
联系方式 Tel	+86-830-6681330	申请日期 Date	July 19, 2021
伦理审查及修改意见<br>Ethical review and comments	☐批准：Approval<br>☐不批准：Disapproval<br>☐作必要修正后同意：Approval after necessary correction		
主审委员（签名）<br>Chairman of the ethics committee	[illegible]	审查日期<br>Date	2021.7.19
审查结果（盖章有效）<br>Evaluation Comments (Seal)<br>泸州市人民医院 医学伦理委员会	经我院医学伦理委员会审查，本项目符合医学伦理原则和赫尔辛基宣言的基本要求，研究设计具有科学根据，没有给受试者带来不必要的危险，人类组织提取的途径是规范的，对受试者的安全和隐私给予了最大限度的保护。同意该项目申报。<br>After review by our medical ethics committee, this project conforms to the basic requirements of medical ethical principles and the Declaration of Helsinki. The research design has a scientific basis. The study poses no unnecessary risk to the subjects. The security and privacy of the subjects are protected to the maximum extent possible. The application for the project is agreed.		
