# Supplementary material for: Pulmonary papillary adenoma with malignant potential: a case report and literature review
Source: Diagn Pathol. 2022 Oct 13;17:81. doi: 10.1186/s13000-022-01259-8 (PMC9563795; doi:10.1186/s13000-022-01259-8)
Supplement: Supplementary file 2 — Supplementary Material 2 [file 13000_2022_1259_MOESM2_ESM.pdf]

BioMed Central

Consent form

I ……[illegible]…… [Name] give my consent for information about myself/my child or ward/my relative (circle as appropriate) to be published in …Diagnostic Pathology. Submission ID: a08f8734-8200-49b9-9cd9-5acf10650Se4. …Corresponding author: FENG Junjian. E-mail: 4994200b4@qq.com…… [Name of journal, manuscript number and corresponding author].

I understand that the information will be published without my/my child or ward's/my relative's (circle as appropriate) name attached, but that full anonymity cannot be guaranteed.
I understand that the text and any pictures or videos published in the article will be freely available on the internet and may be seen by the general public. The pictures, videos and text may also appear on other websites or in print, may be translated into other languages or used for commercial purposes.
I have been offered the opportunity to read the manuscript.

Signing this consent form does not remove my rights to privacy.

Name……[illegible]……

Date……2021.7.15……

Signed……[illegible]……

Author name……[illegible]……

Date……2021.7.15……

Signed……[illegible]……

Please keep this consent form in the patient's case files. The manuscript reporting this patient's details should state that 'Written informed consent for publication of their clinical details and/or clinical images was obtained from the patient/parent/guardian/ relative of the patient. A copy of the consent form is available for review by the Editor of this journal.
